# Does diverse staff and skill mix of teams impact quality of care in long-term elderly health care? An exploratory case study

**DOI:** 10.1186/s12913-018-3812-4

**Published:** 2018-12-20

**Authors:** L. Koopmans, N. Damen, C. Wagner

**Affiliations:** 1 Netherlands Institute for Health Services Research (NIVEL), Utrecht, The Netherlands, currently working at TNO Healthy Living, Leiden, TNO Healthy Living, P.O. Box 3005, NL 2301 DA Leiden, The Netherlands; 2 Netherlands Institute for Health Services Research (NIVEL), Utrecht, The Netherlands, currently working at Elisabeth-TweeSteden Ziekenhuis (ETZ), Tilburg, The Netherlands; 3 Netherlands Institute for Health Services Research (NIVEL), Utrecht, The Netherlands; 4 Department of Public and Occupational Health, EMGO Institute for Health and Care Research, VU University Medical Center, Amsterdam, The Netherlands

**Keywords:** Elderly, Health care reform, Health care quality, Access, And evaluation, Health personnel, Long-term care, Patient care team, Personnel management, Qualitative research

## Abstract

**Objectives:**

Many European countries face challenges in long-term care for older people, such as the growing number of older people requiring care, the increasing complexity of their health care problems, and a decreasing workforce that is inadequately prepared. Optimizing the staff and skill mix of health care teams may offer part of the solution for these challenges. The aim of this study was to obtain insight into the development of teams in terms of staff and skill mix, and the influence of staff and skill mix on quality of care, quality of life, and job satisfaction.

**Methods:**

Seven teams in elderly care in the Netherlands participated in this exploratory case study. From April 2013 to January 2015, a researcher followed the development of the teams, performed observations at the workplace and held interviews with team members, team captains, and (representatives of) clients. Data-analyses were carried out in MAXQDA 11, by coding interviews and analyzing themes.

**Results:**

During the project, almost all teams became more diverse in terms of staff and skill mix. In general, there was a trend towards adding (more) higher-qualified health care workers (e.g. nurse) to the team, increasing communication with other disciplines, and enhancing skills of lower-qualified team members. A more diverse staff and skill mix had a positive effect on quality of care and quality of life of clients, and on job satisfaction, but only under certain contextual conditions. Important contextual conditions for successful functioning of a diverse team were a shared view of care by all team members, good communication, autonomy for professionals, and a safe team culture.

**Conclusion:**

A more diverse staff and skill mix, in combination with positive contextual conditions, can result in improved quality of care, quality of life, and job satisfaction. However, a “one size fits all” blueprint for the optimal staff and skill mix, that suits each team and organization, does not exist. This depends on the context, and should be based on the needs of the clients and possible future changes in these needs.

## Background

Many European countries currently face challenges in long-term care for older people, such as the growing number of older people requiring care, the increasing complexity of their health care problems, and a decreasing workforce that is inadequately prepared.

### An increasing number of older people requiring complex care

In the Netherlands, there are currently three million older people (aged 65+). This is 18% of the total Dutch population. Between 500.000 and 700.000 are classified as frail elderly, most of them living at home. Between 100.000 and 200.000 older people are living in retirement homes or nursing homes. Retirement homes offer more complex medical care than nursing homes in The Netherlands. The number of older people will continue to increase in coming decades. By the year 2040, it is expected that there will be 4,8 million older people in the Netherlands (26% of the total Dutch population), a great number of them over 80 years old [1]. The Dutch government is aiming for older people to live at home for as long as possible, supported by their social network and care at home [2]. This means that care at home becomes more complex, and also that when people do go into a retirement or nursing home, they require more complex care than before.

Thus, in addition to the growing number of older people requiring care, the complexity of their health care problems increases. The number of people with chronic diseases (such as diabetes mellitus, coronary heart disease, or chronic obstructive pulmonary disease) is growing [3]. In addition, the number of older people with dementia will grow due to higher life expectancies [4]. The World Health Organization [5] warns that the world is faced with a massive and growing burden of chronic diseases, which are among the world’s leading causes of death and disability, and trends indicate that they are likely to become even more acute over the next decade.

### A decreasing workforce that is inadequately prepared

Many countries are faced with a decreasing number of health care workers. For example, the numbers of medical graduates entering primary care specialties such as general internal medicine, family medicine, or geriatrics are decreasing in the United States [6]. Also, there are concerns in many European countries about shortages of doctors and nurses [7]. According to the Organisation for Economic Co-operation and Development (OECD), these concerns are likely to intensify in the future as the demand for nurses continues to increase and a wave of retirements among nurses is caused by the ageing of the “baby boomer” generation [7].

Finally, the current workforce is not adequately prepared for the growing burden of chronic diseases [5]. In The Netherlands, for example, most teams in retirement homes and nursing homes traditionally consist of lower-qualified health care workers (e.g., nursing assistants). The qualification level of these employees is generally too low to deal with the increasing complexity of care demands [8]. The Dutch government therefore chooses to invest in recruiting new health care personnel, professional development and education of existing personnel, and more autonomy for health care professionals [9]. It is clear that the growing number of older people requiring care, and the increasing complexity of their health care problems, requires the use of a diverse, sufficiently knowledgeable, and well-equipped health care team [10].

### Staff and skill mix as a solution?

Changing the staff and skill mix of health care teams is a strategy for improving the effectiveness and efficiency of health care [11, 12], and may offer part of the solution for the challenges that long-term elderly care faces. Previous research has shown that quality of care improves when employees within a health care team differ in terms of education, expertise, and competencies [10, 13, 14]. For example, a review by Aiken and colleagues [15] showed that a richer nurse skill mix (a higher percentage of professional nurses among all nursing personnel) was associated with better quality of care, higher safety, and higher hospital ratings from patients. Dubois and Singh [14] also reported in their literature review that a rich mix of qualified personnel with advanced degrees or specialty certifications is associated with better clinical outcomes, such as lower patient mortality rates and fewer home visits in several observational studies [16–18]. Research by Lemieux-Charles and McGuire [19] showed that the type and diversity of clinical expertise involved in team decision making largely accounted for improvements in patient care and organizational effectiveness. In addition, several empirical studies and systematic reviews supported the hypothesis that a higher registered nurse staff mix results in better quality of care e.g. [17, 20–22]. Thus, a diverse staff and skill mix of health care teams, may offer a good solution to provide and maintain high quality of care for the elderly. However, it is currently unclear what this staff and skill mix should look like.

In order to obtain insight into an optimal staff and skill mix of teams in long-term elderly health care in the Netherlands, the project “Living Labs in Elderly Care” was initiated. In this project, seven teams in different health care settings for the elderly in The Netherlands were given the freedom to learn about and explore their optimal staff and skill mix. The aim of this paper is to obtain insight into the development of teams in terms of staff and skill mix, the contextual conditions under which a diverse team is successful, and the influence of staff and skill mix on quality of care, quality of life, and job satisfaction.

## Methods

### Context: Living labs in elderly care

The project “Living Labs in Elderly Care” (“Proeftuinen Ouderenzorg” in Dutch) was initiated in 2012 by the Professional Association of Dutch Nurses (V&VN) and funded by the Dutch Ministry of Health, Welfare and Sport (VWS). The goal of the project was to obtain insight into an optimal staff and skill mix of teams in long-term elderly care. Thus, the goal of the project was not to obtain specific changes within the team, but rather to facilitate the teams in their learning process. A guide from V&VN had regular meetings with each team manager (roughly every three months), to support the manager in guiding the team learning process, to reflect on the developments so far, and to discuss possible future developments.

Seven “Living Labs” were recruited by V&VN. Two of the teams worked in a retirement home, three teams worked in a nursing home, and two teams provided care at home for older people in The Netherlands. Retirement homes and nursing homes both provide accommodation, supervision from staff 24 h a day, assistance with activities of daily living (such as meals and help with personal care needs), but nursing homes provide care for people with more complex needs and those who need regular nursing interventions. If older people can stay at home safely and independently, care at home is encouraged in The Netherlands, as a means of ensuring that older people’s personal care needs are received.

### Study description

The Living Labs ran for one years and ten months (from April 2013 to January 2015). During these 22 months, they were given the freedom to learn about and explore their optimal staff and skill mix. The interdisciplinary team development process was self-directed. There were no demands or restrictions on staff and skill mix. The Living Labs were responsible for their own development and goals, and had to finance changes in staff and skill mix themselves. The start situation of each team is described in Table 1.

**Table 1:** Data on numbers of staff mix and skill mix per team

Team	Start situation	Changes in staff mix	Changes in skill mix
1 (retirement home)	31 team members, mainly nursing assistants level 1 and 2.	− Educating nursing assistants from level 1 to level 2. − Adding 3 nurses (level 4). − Tighter collaboration with other disciplines and clients’ family	- Role enhancement - Role enlargement
2 (care at home)	8 team members: 5 nursing assistants level 2 and 3, 3 nurses level 4.	− Nursing assistants level 1 and 2 gone. Adding 3 nursing assistants level 3.	- Role enhancement - Role enlargement
3 (nursing home)	30 team members: nursing assistants level 2 or 3, and nurses (level 4).	− Adding 2 nurses (level 5). − Educating nursing assistants from level 2 to level 3 − Tighter collaborationwith other disciplines and clients’ family	- Role enhancement - Role enlargement
4 (care at home)	14 team members: 1 nurse (level 4), 8 nursing assistants level 2 and 3, 3 casemanagers, 1 geriatric specialist, 1 psychologist.	− No change in staff mix, but the team members split from 1 into 2 teams. − Tighter collaboration with other disciplines and clients’ family.	- Role enhancement - Role enlargement
5 (nursing home)	51 team members: all nursing assistants level 3.	− Diversifying the team by downgrading nursing assistants to level 1 and 2, by hiring group assistants, and by letting go of some nursing assistants level 3. − Adding nurses (level 4 and 5). − Tighter collaboration with other disciplines and clients’ family.	- Role enhancement - Role enlargement
6 (nursing home)	29 team members: mainly nursing assistants level 2 and 3.	− Adding 4 nurses (level 4). − Educating nursing assistants from level 2 to level 3. − Tighter collaboration with other disciplines.	- Role enhancement - Role enlargement
7 (retirement home)	36 team members: nursing assistants level 1, 2 and 3.	− Adding 2 nurses (level 4). − Tighter collaboration with other disciplines and clients’ family.	− Role enhancement − Role enlargement

### Data collection

As part of the “Living Labs in Elderly Care”, a researcher followed the developments of the Living Labs in creating a more diverse team in terms of staff and skill mix, and examined the effects on quality of care, quality of life, and job satisfaction. Qualitative data were collected at three moments in time: June–August 2013 (T0), June–August 2014 (T1), and December 2014–January 2015 (T2).

Three types of data were collected, namely 1) observations at the workplace, 2) interviews with team captains, team members and, if possible, interviews with clients or their representatives, and 3) notes of team meetings and meetings between the team manager and the guide from V&VN. All team members were informed of the project beforehand by the team captain through e-mail and during team meetings.

Observations at the workplace were made in order to obtain insight into daily work activities and way of working. At each measurement point (T0, T1, T2), observations were made on one day, three times a day, with the systematic observation list Living Environment [23]. This list is used to observe the approach of staff towards clients, whether meaningful activities are offered to clients, the amount of autonomy that is offered to clients, safety in the living environment, and interaction between staff and clients. No specific team members were selected for the observations, but rather the daily work activities and way of working of all the staff that happened to be working were observed on the day the researcher visited. The presence of the researcher was explained to the team members and they could agree or object to being observed in their daily work activities and way of working.

Interviews with team captains and team members were held in order to obtain insight into the current situation, developments within the Living Lab, and experienced quality of care, quality of life of clients, and job satisfaction. At each measurement point, an interview with the team captain and three to five team members (individually) took place. The team captain selected the team members to be interviewed, based on their availability the day that the researcher visited. The researcher made sure that team members with different qualification levels were selected for the interviews. A total of 98 individual interviews were held. In addition, at T2, a total of 11 individual interviews with clients or their representatives were held in order to obtain insight into quality of care and quality of life of clients. The interviews were semi-structured, and were carried out according to a topic list. The interviews were mostly face-to-face, or sometimes via telephone. Team members were informed of the purpose of the interview, and assured that their participation was anonymous and voluntary, and that they could break off the interview at any time. Oral consent from the participant was obtained to record the interview on a voice recorder. The recordings were transcribed in Word after the interview. In addition, relevant Word documents of team meetings and meetings between the team manager and the guide were collected throughout the project.

### Data analysis

Qualitative data-analyses were carried out in MAXQDA 11, by open and thematic coding of the data and analyzing themes. Data from all sources (observations, interviews, notes) were brought together in MAXQDA 11 as text files. Next, the text files were systematically interpreted in light of the research questions [24]. This was done by coding passages of the texts that were relevant for the research questions. As relevant passages in the texts came up, codes in the MAXQDA code system were created. If a relevant passage did not fit with an already created code, a new code was created. In order to adhere to the exploratory nature of the study, data of the first measurement moment were broadly coded by two independent researchers (LK and ND) and the guide, after which consensus on relevant themes and subthemes of the study was reached during a consensus meeting. This resulted in five main themes, and 12 subthemes that fall under the main themes (see Table 2). These themes and subthemes formed the structure of the “coding system” in MAXQDA. The themes and subthemes guided the interview protocol and the data coding of the second and third measurement moment. Data from the second and third measurement moment were coded by three researchers (LK, ND and LvdS), and again consensus was reached during a consensus meeting.

**Table 2:** Themes and subthemes that emerged from the qualitative data analyses

Main theme	Subtheme	Subtheme	Subtheme	Subtheme
Staff and skill mix	Qualification level	Competencies		
Communication	Collaboration	Coordination		
View of care	Care for the individual client	Role of informal caregivers		
Autonomy	Professional conduct	Getting and using professional autonomy		
Team culture	Role of the team captain/leader	Development and learning	Willingness to change	Ability to change

Ethical approval for this research study was given by VU University Medical Center, Amsterdam, The Netherlands (reference number 2014.494).

## Results

### Changes in staff and skill mix

At the start of the project, team sizes ranged from eight to 51 team members, and included for example group assistants, nursing assistants (qualification level 1, 2 or 3), nurses (qualification level 4 or 5), team captains, psychologists, and physiotherapists. During the project, all teams became more diverse in terms of staff and skill mix. Decisions regarding changes in staff and skill mix were made by management, sometimes in consultation with team captains and/or team members. All changes were made with the goal of improving quality of care, but of course had to be institutionally and financially possible.

Changes in *staff mix* mainly focused on including different job qualifications within the team and on obtaining a more multidisciplinary mix within the team, for example by:

- Educating existing personnel so that they obtained a higher qualification level, in order to cope with the increasing complexity of their clients’ health care problems.
- Adding one or more higher-qualified health care workers (e.g. a nurse or specialist nurse) to the team, in order to cope with the increasing complexity of their clients’ health care problems.
- Adding lower-qualified health care workers to the team (e.g. a nursing assistant level 2), in order to ensure personal attention for clients.
- Seeking tighter collaboration with other disciplines (e.g. a general practitioner, psychologist or physiotherapist), so the team became multidisciplinary.
- Seeking tighter collaboration with clients’ family or other representatives, so they were seen as part of the team that cares for the client.

*“Higher-qualified nurses have a good overview of the department. They are able to have an overview of 30 clients and remember the most important things that need to be done. They are able to focus, adequately share information with colleagues and other disciplines”*

~ Team captain, team 1.

Changes in *skill mix* mainly focused on skills that a higher-qualified health care worker would bring, and on role enhancement and/or role enlargement of employees. For example:

- Higher-qualified health care workers (e.g. a nurse or specialist nurse) were thought to add coaching, coordinating, and signaling competencies to the team.
- The role of existing team members was enhanced (role enhancement). For example, team members were trained in their knowledge of and dealing with older people with dementia, or trained to coordinate care.
- The role of existing team members was enlarged (role enlargement). For example, team members were trained in their knowledge of and dealing with patient-centered care, in coordinating and coaching other team members, in collaborating with clients’ family members (or other representatives), and/or the use of new digital information systems at work.

In general, there was a trend towards adding (more) higher-qualified health care workers (e.g. a nurse or specialist nurse) to the team. This was similar across all three settings (retirement home, nursing home, and care at home), as the heath care needs of the people they cared for grew in complexity. Also, across all settings, there was a trend towards increasing collaboration with other disciplines (e.g. general practitioner, psychologist or physiotherapist), as well as with clients and their family (or other representatives).

*“I wish that it was clear to everyone where we are at and especially what the role of the higher-qualified nurse is, because that is sometimes unclear to me. And that the atmosphere at work stays fun, also for the clients. They live here after all. They shouldn’t suffer when there are frictions amongst personnel, because they notice everything. So yes, clarity, it’s mostly clarity… what do we do, what don’t we do?”*

~ Nursing assistant, team 6

Last but not least, the lower-qualified health care workers were given training relating to for example their knowledge of dementia, communication skills, or coordination skills. All changes were made in order for each team to be able to cope with the increasing complexity of clients’ health care problems and their changing health care needs.

*“Some employees experience a year of training as intense. A lot of lower-qualified staff is around 50 years old, and have to go back to school for a year, next to their work. […] Older employees are used to getting classical education. Now they have to make a portfolio, and work with the computer. It sounds strange to us, but there are a lot of employees who have problems with the computer. And who experience that as a barrier. […] What also stops them, is that they don’t get extra salary so why would they do this extra education?”*

~ Team captain, team 6

This current research showed that each job qualification had its own added value.

*“Every team member is important. You always have an added value regardless of which job qualification you’ve got. Diversity can make a good team.”*

~ Team captain, team 3.

Lower-qualified health care workers play an important role in day-to-day care and safety, and social atmosphere for clients. Higher-qualified health care workers play an important role in coordinating care, (early) signaling of health care problems, and coaching of other team members. For an optimal team composition, all job qualifications and associated skills are important.

*“I like the fact that I can approach the higher-qualified nurses with questions, ask them for information, and ideas on how I can do something. She coaches me on the job. I really like that. It is nice to be able to rely on a higher-qualified nurse when something is up with a client.”*

~ Nursing assistant, team 3

*“Many of our employees have downgraded in function, there are a few new employees coming and a few employees are going to leave. We have definitely changed as a team, and team members experience this positively. There is a new way of working. We have a team where all kinds of staff levels are represented, with nursing assistants of level 1, 2 and 3. Level 4 are our higher-educated nurses. In terms of team composition, we are nearly where we wish to be.”*

~ Team captain, team 5

### Experience of the influence of staff and skill mix on quality of care, quality of life, and job satisfaction

#### Quality of care

A more diverse staff and skill mix had a positive effect on quality of care for clients, as reported by the team captains and team members. For example, they indicated that they could help clients more quickly because they worked more efficiently, and that health care professionals were more unified in their care of the client, resulting in less unrest for the client.

In five of the seven participating teams, one or more higher-qualified health care workers (e.g. a nurse or specialist nurse) were added to the team in order to cope with the increasing complexity of their clients’ health care problems. In three of the five teams, team members indicated in interviews that this had a positive influence on quality of care. They perceived that health problems or deteriorating health of clients were detected earlier, and other team members were coached and guided in their day-to-day work.

*“By expanding my team I can better meet the quality of care and quality of life that clients expect. Quality of care has improved and clients notice that. Team members work more precisely. Also, we consult with each other more often and more efficiently, which has a positive effect on the team. Everything is connected.”*

~ Team captain, team 1

“I believe there is added value when there is a higher-qualified nurse in the team. The quality of care is higher, there is earlier signaling of clients’ problematic behavior, of clients dying, of multicomplexity, both physical and cognitive. We also use higher-qualified nurses to improve our team, to support lower-educated nurses.”

~ Team captain, team 6

In two of the five teams, the positive influence of a higher-qualified health care worker on quality of care was not experienced, as appeared from the interviews. The main reason for this was unclarity regarding the role and position of the higher-qualified health care worker within the team. For a successful integration within a team, it is important that it is clear to everyone why the new team member is added, what his or her added value is, which tasks and responsibilities he or she has, and what this means for the tasks and responsibilities of the other team members.

“I do experience that quality of care improves with a higher-qualified nurse in the team, when the team supports the addition of that nurse. But some team members think: do we need this? Do we have to? What is the added value of a higher-qualified nurse in the team? Can’t we do those tasks ourselves? As long as the team members don’t see this added value, adding a higher-qualified nurse gives irritation or friction in the team.”

~ Physiotherapist, team 3

*“We are searching as a team. I came here in a chaotic situation and that chaos is still here, in my experience. That makes it very hard to finds my place in the team. A lot of things are unclear. Materials that we need are not available. And how am I going to fulfil my role as a higher-qualified nurse? My role is still in the starting blocks. We are working on it, but it’s a real searching process.”*

~ Nurse, team 6

#### Quality of life

There were some indications that a more diverse staff and skill mix had a positive effect on quality of life of clients, based on interviews with (representatives of) clients or team members. However, clients’ quality of life was not structurally measured and data were too scarce to draw firm conclusions regarding this outcome. Some team members indicated that the approach towards clients improved because of the influence of higher-qualified health care workers and more time to give personal attention to clients.

*“The last 6 months we have made great strides. Our quality of care has improved. There are hardly any complaints from clients anymore. The approach of team members towards clients is great now. Previously, clients did not want to be cared for by every member of the team, but now they do. Also, there is more time to have a chat with the client.”*

~ Nursing assistant, team 1.

As aforementioned, in the teams, a shift from a task-oriented towards a patient-centered view of care was observed. Team members indicated that they believed this improved clients’ quality of life, for example, because clients were now woken up according to their own preference, instead of by room order, or for example, because family members were more involved.

*“I think clients feel that there is a team of people around them that want to take care of them together, where the client is in the center. They feel that we are in touch with each other. I don’t have to say everything three times before they get it. Their family feels this too. I think clients feel better because of the improved quality of care.”*

~ Casemanager, team 4.

*“My mother is very happy in this care home. She is not lonely. In fact, she is happier now than when she was living on her own. She has a social life again. The employees are very nice and very involved. The quality of the temporary workers, in holiday periods, is sometimes less. They often don’t know who the clients are.”*

~ Family member, team 6.

Nevertheless, a majority of team members as well as clients, or their representatives, indicated that there was a lack of time and/or personnel to give sufficient personal attention to clients. This suggests that there is still room for improvement regarding personal attention for clients in these health care organizations.

*“I wish I could do more for people than just basic care. It’s a piece of wellbeing that I think people are missing. Like today, in the afternoon, I do the administration, bring a round of coffee, bring someone to bed, bring someone to the toilet, and then the afternoon is over. There are no activities, that’s been cut. I think that’s such a shame. I hope that will improve in the future.”*

~ Nursing assistant, team 3.

*“The care for my wife is good. In the beginning, my wife had trouble getting used to the care home, but now she is used to here. The communication is reasonable. Not every employee is as communicative about my wife’s care. I am very satisfied about the nursing assistant who is responsible for my wife’s care. The personal attention that is given to clients differs per employee. Sometimes I feel like they are busier with the administration that with the clients.”*

~ Family member, team 5.

#### Job satisfaction

First and foremost, the interviews and observations showed that the health care professionals in our research were very satisfied, dedicated, and passionate about their job. The influence of a more diverse staff and skill mix on job satisfaction of health care professionals differed. In some teams, changes in the staff and skill mix created unrest and dissatisfaction. Many team members had worked in the team for a long time and some had difficulty adopting a new way of working. Also, the role, tasks, and responsibilities of team members could become unclear after adding new team members.

*“The total package, so not just basic care like washing and dressing, but also the social aspect, is a lot of fun. Sometimes you are in contact with family of the client. You look at the possibilities within the home situation. That can small things. You are very close to someone, in their home situation, and you can make a real difference. That is very precious.”*

~ Nursing assistant, team 2

*“I enjoy going to work. The past half year I enjoyed going to work a bit less because there were hassles, but I said to myself, it is my choice, what I choose to find important. […] The thing with change, I realise, is that change is difficult for people.”*

~ Nursing assistant, team 7

*“There is a lot of negativity in the team. I was on holidays for 1,5 weeks, and I did not look forward to going back to work, because of all the things that have changed again… that is how I feel.”*

~ Nursing assistant, team 7

In other teams, changes in the staff and skill mix had a positive effect on team members’ job satisfaction. In these teams, explicit attention was paid to the reason why the staff and skill mix was changed and what this meant for everyone’s role, tasks, and responsibilities. In these teams, improved collaboration and communication between team members also played an important role in improving job satisfaction. Also, being able to provide higher quality of care had a positive effect on team members’ job satisfaction.

*“We communicate differently with each other now, and we understand each other better. We also dare to talk to each other about things that are not going so well, which really improved the atmosphere in our team. Since a couple of months, I really enjoy going to work again!”*

~ Nurse, team 4

Despite the general high level of job satisfaction, a lot of the health care workers said that they were dissatisfied with the amount of time they had to provide good quality of care for a client and indicated that there were not enough staff to handle the workload.

*“I enjoy going to work. I chose this profession because I like to work with elderly people. I would like time to give more personal attention to clients.”*

*~ Nursing assistant, team 1.*

*“The work atmosphere is very pleasant and I always really enjoy going to work. However, because there are so few personnel, the work pressure gets higher and higher and there is less time to do fun things with clients.”*

~ Nursing assistant, team 3

*“Caring for older people is very special. They deserve good care. However, we have to spend a lot of time behind the computer, filling in forms. We forget what it’s all about: the older, care-needing individual.”*

~ Nursing assistant, team 5

### Contextual conditions

For successful functioning within the diverse teams, four important contextual conditions were observed within all teams, namely good communication, a shared view of care, autonomy for professionals, and a safe team culture. These contextual conditions were important across all three settings (retirement home, nursing home and care at home). Thus, the effects of staff and skill mix were independent of the setting, but rather depended on whether the right contextual conditions were present or not.

Concerning communication, the way that new team members were introduced within the team, was important. In successful teams it was clear *why* the new team member was added, *what* his or her role and responsibilities were, and what this meant for the role and responsibilities of the other team members. In addition, it was important that team members got to know each other and each other’s roles and responsibilities, and to have good communication structures (e.g. team meetings and digital communication systems).

*“There is better communication, team members more openly talk to each other, and not just during official meetings, but also during the workday. And not just about care for the client, but also getting to know each other’s’ area of expertise, what they do during a workday, and how they can use each other’s’ expertise. That kind of dialogue was never there before. It sounds very black and white, but I think that is a real difference.”*

~ Nurse, team 4

In successful teams, team members tended to have a similar view of care and explicitly discussed this. In all teams, a shift from a task-oriented towards a patient-centered view of care was observed. In a patient-centered view of care, the needs and wishes of clients are central, and clients, their family, and other informal caregivers are more involved in the provided care [25]. However, many team members indicated that they sometimes found it difficult to translate a patient-centered view of care into their day-to-day work (e.g. how to actively involve clients and their families in daily routine).

“Satisfied clients is my most important goal of the day. That is where the change is going, the change I’ve been seeing in this profession as an “older” employee. The change from “the doctor or nurse will tell you what’s good for you and everything is decided for you” to more autonomy for clients. I am all for this, but sometimes it’s difficult to know how we can incorporate this autonomy for clients into the daily routine.”

~ Nursing assistant, team 3

Autonomy for professionals played an important role in successfully creating a diverse team. In order to successfully introduce more autonomy for team members, the team captain must give team members independence, and team members must take this independence. However, many team members need to get used to working with more autonomy, and not all team members are willing and/or able to take on more professional freedom. Higher-qualified health care workers can play an important role in successfully introducing more autonomy within the team, by coaching and guiding other team members.

“Team members do see themselves as a professional, but I believe that it’s possible to get much more out of this, to make them even more aware that they are a professional. For example, team members feel very differently about our quality register. Some team members love it, and experience it as part of their professional development. Others think it’s nonsense, and experience it as another task to do.”

~ Nurse, team 5

Finally, a safe team culture was an important success factor for changes within the team. Team members should be given freedom and time to reflect on, think about and discuss their current and optimal team composition in the light of their clients’ needs, and possible future changes in these needs. Team members need to be involved in team changes, in order to be willing to change.

*“The largest insight for me from Living Labs in Elderly Care was: ‘Give people space so they can learn’. If we do not get and take that space, then we cannot learn.”*

~ Team captain, team 3

*“Some team members noticeably find it very difficult to deal with change. You hear a lot of: ‘yes but this, yes but that…’ I find that very disappointing. […] Change is always difficult for some people.”*

~ Nursing assistant, team 7

## Discussion

### Effects of staff and skill mix

The current study showed that a more diverse staff and skill mix, in combination with positive contextual conditions, can have a positive influence on quality of care, quality of life, and job satisfaction. For example, because of a more diverse staff and skill mix and improved communication, teams worked more efficiently, deteriorating health of clients was detected earlier, and personal attention for clients increased. On the other hand, when contextual conditions were negative, or not explicitly addressed, changes in the staff and skill mix could lead to role and task unclarity for health care professionals and decreased job satisfaction. For example, when a higher-qualified health care worker was added to the team, without a clear role and position, this could cause turmoil within the team and lead to lower job satisfaction. This finding could be explained in light of Self-Determination Theory [26].

Central to Self-Determination Theory is the idea that people have three basic psychological needs: autonomy, competence and relatedness. Regarding autonomy, the distinction between autonomous motivation and controlled motivation is very important [27]. In the current study, when team members felt that they had a say in the team changes (autonomous motivation), they felt intrinsically motivated and experienced higher commitment to the team and higher job satisfaction. On the other hand, when team members felt that team changes were externally imposed upon them and they had no say in the matter (controlled motivation), they acted with a sense of pressure and felt a lack of motivation and satisfaction. Based on the current research, an autonomy supportive working environment seems very important for successful creation and functioning of teams with a diverse staff & skill mix.

The finding that a diverse staff and skill mix of health care teams can positively affect quality of care is in line with previous research e.g. [14, 15, 19]. For example, our finding that including higher qualified staff in the team positively affects the quality of care is in line with Aiken and colleagues [15]. The findings support the fact that teamwork is becoming more and more important in health care [19]. However, our study shows that diversifying a team has to be set up carefully in order to avoid negative impacts.

The optimal staff and skill mix should be based on the needs of the clients and possible future changes in these needs. However, the Dutch Health Care Inspectorate found that in more than half of the retirement homes or nursing homes they visited in the Netherlands, the quantity and expertise of personnel was not fitted to the health care needs of clients [28]. Paying attention to this team-client fit is essential, as an optimal fit between the staff and skill mix of the health care team health and the care needs of the clients is a prerequisite for safe and good care.

In recent years, in the Netherlands there is a trend to add (more) higher-qualified health care workers to health care teams in order to cope with increasing complexity of health care problems of the elderly. In a systematic review, Backhaus et al. [29] found no consistent relationship between nurse staffing and quality of care in nursing homes. Higher staffing levels were associated with better as well as lower quality of care indicators. The current study provided qualitative evidence that adding nurses to health care teams in elderly care can have a positive effect on quality of care and quality of life of clients (e.g., early detection of deterioration; coaching and training of other team members), when the role and task of the nurse is clearly defined and communicated. This may be a first step towards gaining insight into the effect of adding (more) higher-qualified health care workers to health care teams in long-term elderly care, and obtaining insight into important conditions for successful integration of these new team members.

The current study emphasizes that it is not only important to look at staff qualification levels within a team, but also at skill mix within a team. Dubois and Singh [14] found that previous research tended to focus solely on staff mix, i.e. achieving a specific mix of different types of personnel. However, organizations should also consider the skills of their workers, and a good mix of these skills, to create a diverse team that is tailored to the needs of the clients.

### Contextual conditions

The current study showed that a diverse staff and skill mix does not automatically result in higher quality of care for patients and higher job satisfaction for employees. Teamwork is complex and many factors are important in realizing the full potential of teams in healthcare. The fact that staff and skill mix cannot be considered in isolation from the context is in line with previous research. For example, Xyrichis and Lowton [30] highlighted the importance of team structure (such as team premises and organisational support) and team processes (such as team meetings and audits) in teamwork. In addition, Dubois and Singh [14] highlight the importance of organisational factors, such as level of autonomy and development opportunities, in creating a fully efficient and effective workforce. The current study highlights the importance of good communication between team members, a shared view of care, professional autonomy, and team culture. An autonomy supportive work environment and explicit attention for teamwork skills seem very important in order to achieve effective functioning of a diverse team.

### Strengths and limitations of this study

This study is one of the first to examine staff and skill mix in long-term elderly care. Most research on staff and skill mix of health care teams focused on the hospital setting, which has a very different way of working and client population than in long-term elderly care. As long-term elderly care (both “care at home” and nursing home care) will become increasingly important in the future, it is important to examine the effects of staff and skill mix on quality of care in long-term elderly care. Second, a strength of the current study is its exploratory nature. This facilitated intrinsic motivation to change within the teams, and allowed the researchers to study the “natural course” of the teams. No predefined staff and skill mix was specified, and organizations and teams were free to change their staff and skill mix according to their own needs and possibilities. Participants indicated that this freedom allowed them to reflect upon their current, and optimal, team composition in the light of their clients’ needs, and possible future changes in these needs, and paved the way to make improvements in their quality of care. In psychological theory, it is well known that intrinsic motivation is an important factor for sustainable behavioral change e.g. [31]. In the current study, the reason (‘why’) for changing staff and skill mix was very important for intrinsic motivation within individual team members.

A limitation of the current study was that data on quality of care, quality of life, and job satisfaction were solely qualitative. Thus, data may be influenced by subjective experiences of the researchers, and the team captains and team members. The idea of the study was to use quantitative data held by health care organizations, and that they would provide this data to the researchers. However, during the course of the study, it appeared that health care organizations either did not collect this information or did not have this information available at the team level (but only at the organizational level). Second, quality of care and quality of life of clients was mainly measured via interviews with health care professionals. Thus, their perceptions of quality of care and quality of life were used as an outcome measure, instead of the clients’ own perception of the quality of care and quality of life. When possible, interviews with clients or their representatives were held. However, many health care professionals indicated that it was difficult to assess quality of care and quality of life directly from the clients, as the clients had severe cognitive limitations (e.g. severe dementia). Another limitation of the current study may be that the managers of the participating health care organizations had a positive attitude toward changing the staff and skill mix (and in particular towards adding higher-qualified health care workers). In health care organizations that had a negative attitude toward changing the staff and skill mix, positive effects on quality of care may be harder to achieve.

### Future recommendations

In general, participating as a “living lab” in the project led to positive changes in the teams, such as reconsidering or aligning the vision of care, improving communication amongst team members, and getting to know each other. The freedom and time that allowed teams to learn and explore was an important success factor. The concept of “living labs” can be used as a method for teams to deliver more patient-centered care and to adapt their staff and skill mix to the needs of their clients. This concept suits the concept of “organizational learning”, which has been suggested as a potential way to meet the increasing demands and challenges within the health care industry [32, 33]. In order to have successful living labs, an autonomy supportive working environment seems very important, in which team members are involved in the process of team change, so that intrinsic motivation and commitment to the team changes is created. Team captains should be supported by a guide in leading the process of change and showing effective leadership. We also recommend that it is beneficial for a small number of teams to learn together and from each other, in a safe and inspiring learning environment If possible, short-term and long-term effects of staff and skill mix changes on quality of care, quality of life, and job satisfaction should be examined. Future research should also further examine the added value of each staff qualification level, and especially that of higher-qualified nurses, on quality of care for older people.

## Conclusion

An optimal staff and skill mix, that is fitted to the needs of the clients, may offer part of the solution for the challenges that long-term care for older people currently faces. However, a “one size fits all” blueprint for the optimal staff and skill mix, that suits each team and organization, does not exist. The optimal staff and skill mix should be based on the needs of the clients and possible future changes in these needs. This study provides insight into what can be done to better fit the staff and skill mix to clients’ health care needs. It also provides the insight that a successful change the staff and skill mix of teams does not come easily. Regardless of the care setting, creating positive contextual conditions (good communication, a shared view of care, autonomy for professionals, and a safe team culture) are essential to make varied teams work.

## Abbreviations

NIVEL: Netherlands Institute for Health Services Research
OECD: Organisation for Economic Co-operation and Development
V&VN: Professional Association of Dutch Nurses
VWS: Dutch Ministry of Health, Welfare and Sport
